# Influence of age and sex on left ventricular diastolic strain analysis

**DOI:** 10.1007/s10554-018-1480-4

**Published:** 2018-10-30

**Authors:** R. W. J. van Grootel, R. M. Kauling, M. E. Menting, J. McGhie, J. W. Roos-Hesselink, A. E. van den Bosch

**Affiliations:** 1 Department of Cardiology, Erasmus University Medical Center, Doctor Molewaterplein 40, 3015 GD Rotterdam, The Netherlands; 2 Department of Radiology, Erasmus University Medical Center, Doctor Molewaterplein 40, 3015 GD Rotterdam, The Netherlands

**Keywords:** Diastolic function, Speckle tracking, Reference values

## Abstract

Speckle tracking analysis (STE) of the left ventricle offers a new method to assess left ventricular (LV) diastolic function. LV diastolic strain measurements offer a non-invasive, global and direct measure of LV diastolic function. However, there is little data on normal values and the influence of anthropomorphic factors which is crucial in clinical practice for new techniques. The aims of this study were to formulate reference values for LV diastolic strain rate, elucidate effects of age and sex on LV diastolic strain analysis and compare STE measurements with conventional LV diastolic measurements. One-hundred-forty-seven healthy subjects aged 20–72 years (≥ 28 subjects per age decade) were prospectively included (Mean age 44 ± 13.7 years, 50% female) and examined with electrocardiography and 2D-echocardiography, including speckle tracking. Left ventricular peak early diastolic strain rate (Sre) was measured in the apical windows, using STE. Men had significantly lower LV Sre values than women (1.02 ± 0.22 vs. 1.18 ± 0.23, p value < 0.001). Left ventricular Sre was inversely associated with age, with values decreasing with ageing. An inverse relation was also found with blood pressure and body surface area. Linear regression analysis showed that LV Sre was independently associated with both age and sex. A multivariable linear regression analysis for LV Sre with conventional LV diastolic variables accounted for 70.9% of the variation of LV Sre, showing good model performance. Reference values for LV Sre are reported and found to be both age- and sex-dependent. Therefore we recommend age- and sex-specific references values to be used in daily clinical practice.

**Electronic supplementary material:**

The online version of this article (10.1007/s10554-018-1480-4) contains supplementary material, which is available to authorized users.

## Introduction

Left ventricular (LV) diastolic disease is increasingly being recognized as an important cause of LV heart failure. Approximately 40% of the patients present themselves with symptoms of heart failure while having a preserved ejection fraction [1]. Increased LV diastolic stiffness and reduced relaxation are thought to be the main pathophysiologic mechanism. Current guidelines on LV diastolic function recommend the use of several echocardiographic Doppler variables, including Tissue Doppler Imaging (TDI), to assess LV relaxation and predict LV filling pressures [2]. Filling pressures are dependent on loading conditions and myocardial relaxation, both are (indirectly) assessed by measuring the speed with which the myocardium lengthens during early diastole and trans mitral flow velocities. While these conventional variables are a good way to assess LV diastolic function, they do have some limitations. TDI variables are angle-dependent and measure LV relaxation only regionally, early and late mitral flow velocities are dependent on both ventricular and atrial pressure and the enlargement of the left atrium may be present in the absence of LV diastolic dysfunction. Deformation measurements derived from speckle-tracking echocardiography (STE) have been validated in human studies [3–17] and offer advantages over TDI such as angle-independency and presenting global relaxation [2], offering an alternative way to assess LV diastolic dysfunction.

Reference values are of vital importance for clinical practice and possibly even more so for new techniques. A recent study published normal values for LV diastolic peak strain rate, but the influences of age and sex were not elucidated [17]. Therefore the aims of this prospective study were to assess and formulate reference values for LV diastolic strain rate in a healthy cohort of volunteers, to assess the possible effects of baseline characteristics, particularly of age, on diastolic strain rate parameters of the left ventricle, and to investigate correlations between conventional diastolic measurements and LV diastolic function assessed with strain rate analysis.

## Methods

### Study population

For this cross-sectional prospective cohort study, healthy volunteers were enrolled into five age-groups: 20–29, 30–39, 40–49, 50–59 and 60–72 years. Each age group had at least 28 subjects, sex was equally distributed in each group. Subjects were recruited via an advertisement for healthy subjects. Volunteers were examined at the Erasmus MC, Rotterdam and excluded if there was any (prior) cardiovascular disease, systemic disease, cardiac medication or the finding of cardiac abnormalities during examination, including signs of LV hypertrophy such as increased wall thickness. Presence of cardiovascular risk factors was also a reason for exclusion, as was renal dysfunction based on creatinine levels. Furthermore, professional athletes, morbidly obese subjects (> 40 kg/m^2^) and women who were pregnant or had breast implants were excluded. Details have been published earlier [18]. The study was carried out in accordance with the principles of the Declaration of Helsinki and approved by the local medical ethics committee. Informed consent was obtained from all participants.

### Clinical assessment

Subjects completed a questionnaire about medical history and health status and underwent physical examination, venous blood sampling, 12-lead electrocardiography and echocardiography. Physical examination included height, weight, blood pressure, saturation and heart, lungs and abdominal findings.

### Echocardiographic image acquisition

All echocardiographic studies were performed by an experienced sonographer (JM). Two-dimensional greyscale harmonic images were obtained in the left lateral decubitus position using an iE33 or EPIQ7 ultrasound system (Philips Medical Systems, Best, The Netherlands) equipped with a transthoracic broadband X5-1 matrix transducer. Standard apical 4-chamber (A4C), 2-chamber (A2C) and 3-chamber (A3C) views were obtained for strain analysis, with framerates ≥ 50 frames/s, for strain analysis.

### Conventional echocardiographic measurements

The recent guideline for chamber quantification from the American Society of Echocardiography and the European Association of Cardiovascular Imaging were followed for echocardiographic measurements [19]. Similarly, the guidelines from the American Society of Echocardiography and European Association of Cardiovascular Imaging were used to assess LV diastolic function [2]. Left ventricular diastolic function was considered abnormal according to the following variables: septal e′ < 7 cm/s or lateral e′ < 10 cm/s using Tissue Doppler Imaging (TDI), an E/e′-ratio > 14, Left atrial maximum volume > 34 ml/m^2^ or a tricuspid regurgitation (TR) jet velocity > 2.8 m/s. All measurements were done whilst being blinded from the clinical characteristics.

### Speckle tracking analysis

**Fig. 1:**
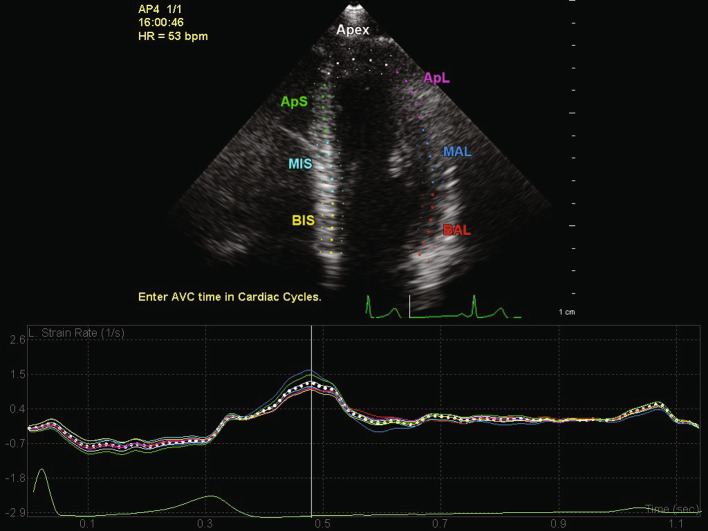
Example of left ventricular (LV) strain measurement in the apical four-chamber view. The colored lines in the graph show LV strain rate measurements for each corresponding segment, the dotted line represents the average strain rate for the all the segments combined

All measurements were performed by a single investigator (RG). The data sets were blinded regarding subject ID. Offline analysis was performed using QLAB 10 (Philips Medical Systems, Best, the Netherlands). Cardiac cycles were defined by the positioning of R-waves, aortic valve closure time was used to define end-systole [20] and obtained by selecting the frame of aortic valve closure on the A3C view. LV strain analysis was performed in the apical four-, three- and two-chamber views (A4C, A3C, A2C). The width of the segments was set to line up with the epicardial and endocardial border, while avoiding the epicardium. The program automatically divided the walls in segments based on the 17 segment model and tracked these on a frame-by-frame basis [21]. When tracking was suboptimal, the borders were adjusted. Segments with persistently inadequate tracking were excluded for analysis. Also if three or more segments were inadequate the measurements were excluded. Peak strain rate was defined as the peak value during early diastole. For each segment the LV early diastolic strain rate peak (LV Sre) was assessed. The average of LV Sre was calculated for each view and for the entire left ventricle by averaging the segmental LV Sre values (Fig. 1).

### Statistical analysis

Normal distribution was checked visually using histograms and with the Shapiro–Wilk test. Depending on data distribution, continuous data are presented as mean ± standard deviation (SD) or median [Q1–Q3]. Categorical data are presented as frequencies and percentages. For comparison the paired *t*-test, Student’s *t*-test, Mann-Whitney-U test, χ^2^-test, Fisher exact test was used as was appropriate. Linear regression analysis was used to assess correlations between baseline characteristics and strain variables. A second model analysis was done to assess correlations between conventional diastolic variables and LV early peak diastolic strain rate. Variables that reached p < 0.001 and did not show collinearity with other variables were included in a multivariable model. In case of collinearity (Pearson r > 0.6), the variable with the highest correlation was included. Statistical analysis was done using the Statistical Package for Social Science version 21 (IBM DPDD Statistics for Windows, Armonk, New York, USA). Statistical test were considered significant with a p value of ≤ 0.05.

## Results

### Study population

Of the 155 eligible volunteers, 147 were included in the study. Reasons for exclusion were breast implants (n = 2), valvular pathology (n = 2), surgically closed duct (n = 1), hypertension (n = 1), morbid obesity (n = 1) and right bundle branch block on ECG (n = 1). Baseline characteristics of the study population per age group are shown in Table 1. Overall, the mean age was 44.6 SD 13.8 years and 50% was female (also equally distributed per age-group). Median systolic and diastolic blood pressure was 126 (116–133) and 80 (75–85) mmHg respectively. Of the 147 volunteers included, 135 (92%) had normal diastolic function and 9 (6%) had an indeterminate diastolic function, according to the current guideline [2].

**Table 1:** Table presenting baseline characteristics

Age (years)	20–29	30–39	40–49	50–59	60–72
	n = 32	n = 28	n = 28	n = 31	n = 28
Female (%)	16 (50%)	24 (50%)	24 (50%)	16 (52%)	14 (50%)
Age (years)	26 ± 3	35 ± 3	44 ± 3	55 ± 3	64 ± 3
Physical examination					
Body mass index (kg/m^2^)	22.3 ± 2.1	24.1 ± 3.4	24.6 ± 3.7	25.4 ± 2.8	25.8 ± 3.1
Body surface area (m^2^)	1.84 ± 0.17	1.89 ± 0.18	1.92 ± 0.22	1.92 ± 0.18	1.90 ± 0.19
Systolic blood pressure	124 ± 13	121 ± 10	123 ± 12	130 ± 15	137 ± 17
Diastolic blood pressure	76 ± 8	78 ± 7	80 ± 10	83 ± 11	83 ± 8
ECG					
Sinus rythm (%)	32 (100%)	28 (100%)	28 (100%)	31 (100%)	28 (100%)
Heart rate (beats per minute)	61 ± 11	60 ± 8	61 ± 10	62 ± 9	65 ± 11
QRS duration (ms)	96 ± 8	97 ± 9	97 ± 9	95 ± 10	97 ± 10
Echocardiography, left ventricle					
End-diastolic dimension (mm)	46 ± 3	47 ± 3	45 ± 4	45 ± 5	44 ± 5
End-systolic dimension (mm)	29 ± 3	28 ± 3	28 ± 5	28 ± 4	28 ± 6
End-diastolic volume/BSA (ml/m^2^)	63.9 ± 9.5	63.1 ± 9.9	64.1 ± 10.9	60.5 ± 8.0	60.6 ± 12.2
End-systolic volume/BSA (ml/m^2^)	25.4 ± 5.1	24.7 ± 6.2	26.2 ± 5.5	23.2 ± 5.1	25.0 ± 6.9
Ejection fraction (%)	60.4 ± 3.6	61.4 ± 5.0	59.2 ± 4.5	61.9 ± 5.3	59.2 ± 4.5
Global longitudinal strain (%)	− 20.8 ± 1.9	− 21.3 ± 1.9	− 20.7 ± 2.2	− 21.1 ± 2.4	− 20.0 ± 1.5
E-wave (m/s)	0.80 ± 0.16	0.75 ± 0.16	0.66 ± 0.15	0.65 ± 0.11	0.59 ± 0.13
A-wave (m/s)	0.39 ± 0.14	0.43 ± 0.08	0.47 ± 0.10	0.56 ± 0.12	0.62 ± 0.17
Deceleration time (ms)	177 ± 29	181 ± 32	185 ± 29	194 ± 31	216 ± 64
E′ (cm/s)	12.5 ± 1.8	10.4 ± 1.6	9.2 ± 1.6	8.2 ± 1.8	6.8 ± 1.7
E/A-ratio	2.27 ± 0.77	1.80 ± 0.40	1.43 ± 0.38	1.21 ± 0.34	1.01 ± 0.32
E/E′-ratio	6.45 ± 1.35	7.27 ± 1.47	7.29 ± 1.71	8.11 ± 1.44	9.04 ± 2.42
Left atrial maximum volume (ml/m^2^)	27.8 ± 5.7	28.1 ± 6.6	29.0 ± 9.2	29.4 ± 5.5	30.0 ± 9.1

Values are presented as mean ± SD or n (%)

*BMI* body mass index, *BSA* body surface area, *EDD* end diastolic dimension, *ESD* end systolic dimension, *EDV* end diastolic volume, *ESV* end systolic volume, *EF* ejection fraction, *E* peak mitral inflow velocity at early diastole, *A* peak mitral inflow velocity at late diastole, *E*′ early diastolic annular myocardial velocity, *LAVI* left maximum volume indexed

### Diastolic strain rate

**Fig. 2:**
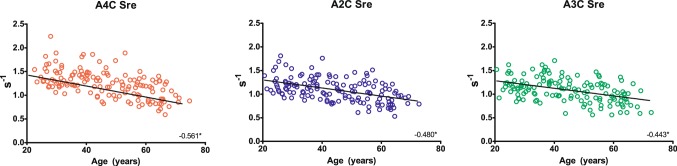
Left ventricular diastolic strain rate for each apical view, showing the correlation with age. In the scatterplot each dot represents a person, for each view a scatterplot was made. The r shows the strength of the correlation which was significant in each view (p < 0.001)

Strain rate analysis was highly feasible, it was possible to measure global strain rate in 98.0% of the patients in the A4C-view, 97.3% of the A2C-views and 95.9% of the A3C-views. Of the entire cohort the mean global LV Sre values of each view were: A4C Sre 1.14 SD 0.29 s^−1^, A2C Sre: 1.09 SD 0.25 s^−1^ and A3C Sre 1.09 SD 0.25 s^−1^. The mean LV global Sre was 1.11 SD 0.24 s^−1^. The LV Sre values per age group are presented in Table 2 and supplemental Table 1. Linear regression analysis with age as a continuous variable showed that every segment was significantly inversely related to age (Fig. 2). Of the three views, LV Sre measured in the A4C view correlated strongest with age (r = − 0.561, p value < 0.001). Besides having the strongest decrease with age, the strain rate values in the different segments of the A4C-view were also the highest.

**Table 2:** Table presenting left ventricular diastolic strain rate per age group

	20–29	30–39	40–49	50–59	60–72	r	p value*
	n = 32	n = 28	n = 28	n = 31	n = 28		
A4C global strain rate (s^−1^)	1.34 ± 0.26	1.27 ± 0.19	1.15 ± 0.27	1.04 ± 0.25	0.89 ± 0.23	− 0.561	< **0.001**
A3C global strain rate (s^−1^)	1.17 ± 0.20	1.23 ± 0.23	1.11 ± 0.24	1.01 ± 0.25	0.91 ± 0.21	− 0.443	< **0.001**
A2C global strain rate (s^−1^)	1.24 ± 0.23	1.18 ± 0.18	1.09 ± 0.23	1.02 ± 0.26	0.90 ± 0.18	− 0.48	< **0.001**
LV global diastolic strain rate (s^−1^)	1.25 ± 0.19	1.23 ± 0.17	1.12 ± 0.22	1.02 ± 0.23	0.90 ± 0.17	− 0.556	< **0.001**

Significant p values are given in bold

*Significancy of linear regression analysis with age (continuous)

*A4C* apical four-chamber, *A3C* apical three-chamber, *A2C* apical two-chamber, *LV* left ventricular

### Influences of baseline characteristics

**Fig. 3:**
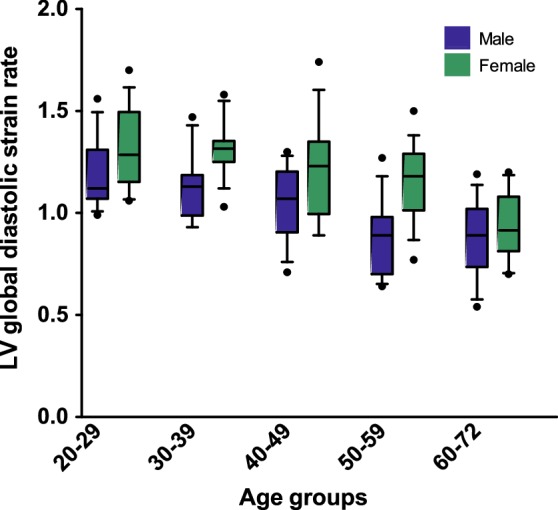
A graph showing left ventricular global diastolic strain rate in boxplots per age decade and stratified for sex. Differences between sex were significant in all age groups, with higher values for the women. Values for left ventricular early diastolic strain rate decrease when age increases

Left ventricular Sre values were sex-dependent, with lower values for males in every segment (Table 3 and Supplemental Table 2). We further explored sex-dependency by also stratifying for age (Fig. 3). This figure shows that values for females were higher across all age groups, and there seems to be a sharper decline in LV diastolic function after the 5th decade.

**Table 3:** Table presenting segmental left ventricular diastolic strain rate per sex

	Female	Male	p value
	n = 74	n = 73	
A4C global strain rate (s^−1^)	1.24 ± 0.30	1.04 ± 0.24	< **0.001**
A3C global strain rate (s^−1^)	1.15 ± 0.24	1.02 ± 0.25	**0.001**
A2C global strain rate (s^−1^)	1.16 ± 0.24	1.02 ± 0.23	**0.001**
LV global diastolic strain rate (s^−1^)	1.18 ± 0.23	1.02 ± 0.22	< **0.001**

Significant p values are given in bold

*A4C* apical four-chamber, *A3C* apical three-chamber, *A2C* apical two-chamber, *LV* left ventricular

Univariable linear regression analysis was performed between baseline characteristics and LV Sre (Table 4). Both a higher systolic and diastolic blood pressure led to a linear decrease in LV Sre values. Lower LV volumes (indexed for BSA) were associated with a higher LV Sre.

**Table 4:** Table showing linear regression analysis

		Left ventricular diastolic strainrate				
		Univariable		Multivariable		
		Pearson’s r	p value	Unstandardized β	95% CI	p value
**Univariable linear regression analysis**						
*Physical examination*						
Female		0.337	< **0.001**			
Age		− 0.556	< **0.001**			
Body mass index		− 0.301	< **0.001**			
Body surface area		− 0.383	< **0.001**			
Systolic blood pressure		− 0.417	< **0.001**			
Diastolic blood pressure		− 0.415	< **0.001**			
*ECG*						
Heart rate		− 0.310	0.713			
QRS duration		− 0.179	**0.032**			
**Stepwise linear regression analysis**						
Age				− 0.010	(− 0.012 to 0.007)	< **0.001**
Model 1	Age			− 0.010	(− 0.012 to − 0.008)	< **0.001**
	Sex			0.171	(0.113–0.230)	< **0.001**
Model 2	Age			− 0.009	(− 0.011 to − 0.007)	< **0.001**
	BSA			− 0.412	(− 0.569 to − 0.255)	< **0.001**
Model 3	Age			− 0.01	(− 0.012 to − 0.007)	< **0.001**
	Sex			0.122	(0.039–0.205)	**0.004**
	BSA			− 0.183	(− 0.401 to 0.034)	0.098
Model 4	Age			− 0.009	(− 0.011 to − 0.007)	< **0.001**
	Sex			0.108	(0.026–0.190)	**0.010**
	BSA			− 0.141	(− 0.357 to 0.076)	0.201
	Systolic blood pressure			− 0.003	(− 0.005 to 0.000)	**0.025**
**Multivariable linear regression analysis**						
*Echocardiography, left ventricle*						
End-diastolic dimension		0.030	0.719			
End-systolic dimension		− 0.097	0.253			
End-diastolic volume/BSA		− 0.203	**0.015**	− 0.001	(− 0.003 to 0.001)	0.360
End-systolic volume/BSA		− 0.378	< **0.001**			
Ejection fraction		0.480	< **0.001**	0.003	(− 0.003 to 0.010)	0.315
Global longitudinal strain		− 0.674	< **0.001**	− 0.054	(− 0.069 to − 0.039)	< **0.001**
E-wave velocity		0.605	< **0.001**	0.264	(0.081–0.448)	**0.005**
A-wave velocity		− 0.296	< **0.001**			
Deceleration time		− 0.368	< **0.001**	0.001	(− 0.001 to 0.000)	0.141
E′		0.617	< **0.001**	0.033	(0.023–0.043)	< **0.001**
E/A-ratio		0.531	< **0.001**			
E/E′-ratio		− 0.177	**0.035**			
Left atrial maximum volume		0.068	0.436			

The first part shows univariable analysis with baseline characteristics, the second part is a stepwise linear regression analysis and the third part presents a multivariable linear regression analysis with conventional left ventricular diastolic indices. Variables that reached p < 0.001 and did not show collinearity with other variables were included in a multivariable model. When there was collinearity, the variable with the strongest correlation was included

Adjusted r^2^ of multivariable model: 0.709

Signicant p values are given in bold

*BSA* body surface area, *E* peak mitral inflow velocity at early diastole, *A* peak mitral inflow velocity at late diastole, *E*′ early diastolic annular myocardial velocity

Table 4 also shows a stepwise multivariable linear regression analysis of baseline characteristics and LV global Sre. This shows that LV global Sre is independently correlated with age, sex and systolic blood pressure.

A second multivariable linear regression analysis was performed (Table 4) which included several conventional LV diastolic variables: E-wave velocity, deceleration time, e′, and also LV end diastolic volume indexed for BSA, LV ejection fraction (EF) and LV global longitudinal strain (GLS). The adjusted r^2^ was 0.709, meaning 70.9% of the variation of LV Sre can be predicted with this model. E-wave velocity and e′ were independently correlated with LV SRe, as were the systolic indices LV EF and LV GLS.

## Discussion

This study demonstrates a clear relation between LV early diastolic peak strain rate and age. There is a clear decrease of LV Sre with ageing, which is especially notable after the fifth decade. The second important finding is that there are significant sex-dependent differences, with males having consistently lower LV Sre values than females.

Reference values are crucial in clinical practice to distinguish normal from abnormal. A previous study determined the lower limit of normal of LV Sre in a large population of healthy subjects [17] and evaluated the added value to conventional assessment of LV diastolic function. The lower limit of normal in their cohort was 1.00 s^−1^, based on their mean value of 1.56–2SD. The mean in our cohort was notably lower, namely 1.10 ± 0.24, which would result in a lower limit of normal of 0.62 s^−1^. A possible explanation for this difference may be the higher mean age of our study population (44.6 SD 13.7 in our study vs. 36.5 SD 12.8 years in the study of Morris et al [17]).

It has been widely recognized that with age, LV diastolic function decreases even in the absence of cardiovascular disease [2]. It considered to be either a part of the ageing process, or as a physiological process, where the left ventricle is becoming slightly stiffer. The added value over for instance Morris et al. [17] is that this study is the first to demonstrate this ageing process is also measurable when assessing LV diastolic function with STE. A closer inspection shows a sharper decrease in LV diastolic function at the onset of the fifth decade, as can be seen in Fig. 1. The combination of a younger study population and this sharper decline at the fifth decade may explain why Morris et al. [17] found no age-dependency. Though no invasive measurements were performed, none of the volunteers had LV diastolic dysfunction according to current guidelines.

Though the current guidelines do not mention differences in LV diastolic function between men and women, we found consistently lower LV diastolic Sre values in men. The difference in mean LV Sre between men and women was 0.16 s^−1^. Marwick et al. reported that sex is independently correlated with LV diastolic Sre [22], and Morris et al. found modestly though significantly lower values in men [17]. These studies all suggest that there is a need for sex-specific LV Sre values.

A few studies have looked at LV Sre in populations with a wide variety of diseases and conditions, but only a few studies have actually looked at LV Sre in a cohort of healthy subjects. Besides age and sex, body size, blood pressure and QRS-duration were correlated with LV diastolic Sre. We also expected heart rate to correlate with LV diastolic function, but this was not the case in our study, most likely due to the lack of variation in heart rate (62 SD 10 bpm in our study population).

The current algorithm to assess LV diastolic function is very good but relies on variables which have some limitations; the early diastolic peak velocity of the mitral annulus assessed with TDI which is angle-dependent, maximum LA volume can be enlarged is in the absence of LV diastolic dysfunction as it is an indirect measure of LV diastolic function, and E- and A-wave velocities are dependent on LV and left atrial filling pressures [2]. This makes them less appropriate for instance in patients with a dilated left ventricle [23], patients with regional dysfunction [24] and patients with atrial fibrillation [25]. In our study population consisting of healthy individuals feasibility of conventional LV diastolic variables was very good. Recently the added value of LV Sre on top of the current algorithm was examined and revealed that LV Sre has indeed added value to diagnose LV diastolic dysfunction [17]. We argue that the use of age- and sex-specific values can improve this algorithm even further.

The results also showed that LV systolic function was independently associated with LV Sre. A better systolic function logically leads to better diastolic function [2] (in the absence of cardiac disease) as this leads to better diastolic function as can be witnessed by an enhanced twisting and untwisting motion of the left ventricle [26].

### Limitations

Between-vendor variability is often mentioned as a limitation for clinical application of strain analysis. In this study, we only used Philips ultrasound equipment and QLAB software. However, after the implementation of the standardization process, the variability of strain measurements in later developed versions of major ultrasound manufacturers have been reduced, making the reported values by us more widely applicable nowadays. Several studies showed that differences between vendors are very small, albeit statistically significant, though one could argue if these differences are clinically relevant [27, 28]. Due to the relatively small sample size, conclusions should be drawn with caution.

## Conclusion

This study presents reference values for LV diastolic early diastolic strain rate peak based on a cohort of healthy individuals with a large variation in age and revealed that LV diastolic function assessed with STE, is age- and sex-dependent. We therefore strongly advise sex- and age-specific reference values for LV diastolic strain measurements with STE in daily clinical practice.

## Electronic supplementary material

Below is the link to the electronic supplementary material.

Supplementary material 1 (DOCX 20 KB)

Supplementary material 2 (DOCX 17 KB)
