# Supplementary material for: Influence of age and sex on left ventricular diastolic strain analysis
Source: Int J Cardiovasc Imaging. 2018 Oct 30;35(3):491–8. doi: 10.1007/s10554-018-1480-4 (PMC6453864; doi:10.1007/s10554-018-1480-4)
Supplement: Supplementary file 1 — Supplementary material 1 (DOCX 20 KB) [file 10554_2018_1480_MOESM1_ESM.docx]

| **Supplemental table 1 - Diastolic strain rate per age group** | | | | | | | |
| --- | --- | --- | --- | --- | --- | --- | --- |
| Age (years) | 20-29 | 30-39 | 40-49 | 50-59 | 60-72 | r | p-value* |
|  | n = 32 | n = 28 | n = 28 | n = 31 | n = 28 |  |  |
| *Apical 4-chamber* |  |  |  |  |  |  |  |
| Basal septal | 1.15 ± 0.24 | 1.07 ± 0.25 | 1.00 ± 0.28 | 0.91 ± 0.23 | 0.72 ± 0.22 | -0.522 | **<0.001** |
| Mid septal | 1.21 ± 0.29 | 1.09 ± 0.25 | 0.91 ± 0.25 | 0.86 ± 0.27 | 0.79 ± 0.27 | -0.519 | **<0.001** |
| Apical septal | 1.54 ± 0.35 | 1.55 ± 0.25 | 1.40 ± 0.36 | 1.27 ± 0.34 | 1.11 ± 0.36 | -0.432 | **<0.001** |
| Apex | 1.46 ± 0.31 | 1.44 ± 0.21 | 1.29 ± 0.37 | 1.13 ± 0.35 | 0.99 ± 0.26 | -0.504 | **<0.001** |
| Apical lateral | 1.38 ± 0.30 | 1.34 ± 0.25 | 1.19 ± 0.41 | 1.05 ± 0.27 | 0.87 ± 0.24 | -0.533 | **<0.001** |
| Mid lateral | 1.37 ± 0.30 | 1.24 ± 0.29 | 1.22 ± 0.28 | 1.08 ± 0.27 | 0.92 ± 0.24 | -0.478 | **<0.001** |
| Basal lateral | 1.26 ± 0.27 | 1.16 ± 0.27 | 1.04 ± 0.30 | 0.97 ± 0.27 | 0.85 ± 0.26 | -0.474 | **<0.001** |
| A4C global strain rate | 1.34 ± 0.26 | 1.27 ± 0.19 | 1.15 ± 0.27 | 1.04 ± 0.25 | 0.89 ± 0.23 | -0.561 | **<0.001** |
| *Apical 3-chamber* |  |  |  |  |  |  |  |
| Basal inferolateral | 1.09 ± 0.26 | 1.07 ± 0.26 | 1.06 ± 0.29 | 0.95 ± 0.32 | 0.89 ± 0.27 | -0.278 | **0.001** |
| Mid inferolateral | 1.16 ± 0.22 | 1.23 ± 0.39 | 1.14 ± 0.26 | 1.02 ± 0.30 | 0.92 ± 0.23 | -0.327 | **<0.001** |
| Apical lateral | 1.26 ± 0.27 | 1.30 ± 0.26 | 1.12 ± 0.29 | 1.01 ± 0.28 | 0.87 ± 0.25 | -0.501 | **<0.001** |
| Apex | 1.31 ± 0.29 | 1.36 ± 0.27 | 1.24 ± 0.32 | 1.06 ± 0.34 | 0.96 ± 0.23 | -0.454 | **<0.001** |
| Apical anterior | 1.39 ± 0.37 | 1.42 ± 0.34 | 1.38 ± 0.38 | 1.12 ± 0.44 | 1.07 ± 0.29 | -0.361 | **<0.001** |
| Mid anteroseptal | 1.07 ± 0.32 | 1.22 ± 0.33 | 0.96 ± 0.30 | 1.02 ± 0.32 | 0.90 ± 0.27 | -0.242 | **0.004** |
| Basal anteroseptal | 0.95 ± 0.23 | 0.98 ± 0.26 | 0.87 ± 0.24 | 0.88 ± 0.23 | 0.75 ± 0.20 | -0.294 | **<0.001** |
| A3C global strain rate | 1.17 ± 0.20 | 1.23 ± 0.23 | 1.11 ±0.24 | 1.01 ± 0.25 | 0.91 ± 0.21 | -0.443 | **<0.001** |
| *Apical 2-chamber* |  |  |  |  |  |  |  |
| Basal inferior | 1.09 ± 0.23 | 1.01 ± 0.23 | 1.00 ± 0.25 | 0.86 ± 0.26 | 0.79 ± 0.20 | -0.423 | **<0.001** |
| Mid inferior | 1.17 ± 0.28 | 0.99 ± 0.30 | 0.96 ± 0.29 | 0.88 ± 0.29 | 0.83 ± 0.24 | -0.378 | **<0.001** |
| Apical inferior | 1.39 ± 0.28 | 1.43 ± 0.27 | 1.25 ± 0.34 | 1.25 ± 0.33 | 1.03 ± 0.27 | -0.399 | **<0.001** |
| Apex | 1.31 ± 0.27 | 1.28 ± 0.24 | 1.16 ± 0.32 | 1.13 ± 0.33 | 0.96 ± 0.22 | -0.4 | **<0.001** |
| Apical anterior | 1.23 ± 0.29 | 1.14 ± 0.28 | 1.08 ± 0.32 | 1.03 ± 0.7 | 0.91 ± 0.24 | -0.345 | **<0.001** |
| Mid anterior | 1.27 ± 0.27 | 1.20 ± 0.25 | 1.12 ± 0.31 | 1.06 ± 0.24 | 0.95 ± 0.27 | -0.382 | **<0.001** |
| Basal anterior | 1.20 ± 0.26 | 1.21 ± 0.30 | 1.08 ± 0.31 | 0.96 ± 0.29 | 0.86 ± 0.25 | -0.416 | **<0.001** |
| A2C global strain rate | 1.24 ± 0.23 | 1.18 ± 0.18 | 1.09 ± 0.23 | 1.02 ± 0.26 | 0.90 ± 0.18 | -0.48 | **<0.001** |
| LV global diastolic strain rate | 1.25 ± 0.19 | 1.23 ± 0.17 | 1.12 ± 0.22 | 1.02 ± 0.23 | 0.90 ± 0.17 | -0.556 | **<0.001** |
