# Supplementary material for: Influence of age and sex on left ventricular diastolic strain analysis
Source: Int J Cardiovasc Imaging. 2018 Oct 30;35(3):491–8. doi: 10.1007/s10554-018-1480-4 (PMC6453864; doi:10.1007/s10554-018-1480-4)
Supplement: Supplementary file 2 — Supplementary material 2 (DOCX 17 KB) [file 10554_2018_1480_MOESM2_ESM.docx]

| **Supplemental table 2 - Diastolic strain rate per sex** | | | |
| --- | --- | --- | --- |
|  | Female | Male | p-value |
|  | n = 74 | n = 73 |  |
| *Apical 4-chamber* |  |  |  |
| Basal septal | 1.06 ± 0.32 | 0.88 ± 0.20 | **<0.001** |
| Mid septal | 1.04 ± 0.31 | 0.91 ± 0.28 | **0.009** |
| Apical septal | 1.47 ± 0.38 | 1.28 ± 0.33 | **0.002** |
| Apex | 1.37 ± 0.34 | 1.15 ± 0.33 | **<0.001** |
| Apical lateral | 1.28 ± 0.36 | 1.06 ± 0.30 | **<0.001** |
| Mid lateral | 1.26 ± 0.33 | 1.07 ± 0.27 | **<0.001** |
| Basal lateral | 1.16 ± 0.30 | 0.95 ± 0.27 | **<0.001** |
| A4C global strain rate | 1.24 ± 0.30 | 1.04 ± 0.24 | **<0.001** |
| *Apical 3-chamber* |  |  |  |
| Basal inferolateral | 1.09 ± 0.28 | 0.94 ± 0.28 | **0.001** |
| Mid inferolateral | 1.15 ± 0.31 | 1.04 ± 0.29 | **0.270** |
| Apical lateral | 1.19 ± 0.31 | 1.05 ± 0.30 | **0.007** |
| Apex | 1.27 ± 0.31 | 1.11 ± 0.33 | **0.004** |
| Apical anterior | 1.35 ± 0.38 | 1.20 ± 0.39 | **0.018** |
| Mid anteroseptal | 1.10 ± 0.35 | 0.98 ± 0.28 | **0.026** |
| Basal anteroseptal | 0.93 ± 0.26 | 0.85 ± 0.22 | **0.037** |
| A3C global strain rate | 1.15 ± 0.24 | 1.02 ± 0.25 | **0.001** |
| *Apical 2-chamber* |  |  |  |
| Basal inferior | 1.00 ± 0.26 | 0.90 ± 0.25 | **0.026** |
| Mid inferior | 1.03 ± 0.33 | 0.91 ± 0.26 | **0.020** |
| Apical inferior | 1.36 ± 0.32 | 1.19 ± 0.32 | **0.002** |
| Apex | 1.25 ± 0.31 | 1.09 ± 0.28 | **0.002** |
| Apical anterior | 1.15 ± 0.34 | 1.02 ± 0.28 | **0.014** |
| Mid anterior | 1.20 ± 0.30 | 1.05 ± 0.25 | **0.001** |
| Basal anterior | 1.13 ± 0.30 | 1.00 ± 0.31 | **0.010** |
| A2C global strain rate | 1.16 ± 0.24 | 1.02 ± 0.23 | **0.001** |
| LV global diastolic strain rate | 1.18 ± 0.23 | 1.02 ± 0.22 | **<0.001** |
